# Comment on ‘Clinical significance of *BRAF* non-V600E mutations on the therapeutic effects of anti-EGFR monoclonal antibody treatment in patients with pretreated metastatic colorectal cancer: the Biomarker Research for anti-EGFR monoclonal Antibodies by Comprehensive Cancer genomics (BREAC) study’

**DOI:** 10.1038/s41416-018-0012-2

**Published:** 2018-03-22

**Authors:** Matthew Dankner, April A. N. Rose

**Affiliations:** 1 Goodman Cancer Research Centre, McGill University, Montreal, QC Canada; 2 Department of Medicine, McGill University, Montreal, QC Canada; 3 Department of Medicine, Division of Medical Oncology, University of Toronto, Toronto, ON Canada

**Keywords:** Targeted therapies, Colon cancer

We read, with great interest, the manuscript published in a recent issue of *British Journal of Cancer*, entitled “Clinical significance of *BRAF* non-V600E mutations on the therapeutic effects of anti-EGFR monoclonal antibody treatment in patients with pretreated metastatic colorectal cancer: the Biomarker Research for anti-EGFR monoclonal Antibodies by Comprehensive Cancer genomics (BREAC) study”.^1^ In this publication, Shinozaki et al. provide preliminary evidence that patients with BRAF non-V600E mutant metastatic colorectal cancers (mCRC) may be resistant to epidermal growth factor receptor (EGFR) inhibition. The results from the retrospective BREAC study are consistent with the emerging paradigm that any activating MAPK mutation (KRAS, NRAS, BRAF V600E) is sufficient to promote intrinsic resistance to EGFR inhibitors.^2–4^ Conversely, these data represent a stark contrast to a recent retrospective analysis of clinical outcomes for mCRC patients with non-V600 BRAF mutations.^5^ Jones et al. have demonstrated that non-V600 BRAF mutant mCRC represents a clinically distinct molecular subtype, which is associated with significantly longer overall survival (OS) compared to mCRC patients with BRAF V600E mutations. Herein, we will explore some possible explanations for the discrepancy in findings between these two recent studies.

Shinozaki et al. performed retrospective analyses on an “inference cohort” (*n* = 150) of 403 mCRC patients. In the BREAC study, all patients had received multiple lines of systemic therapy, and therefore represented a heavily pre-treated population compared to the patients analyzed in Jones et al. wherein survival was calculated from the time of first diagnosis of metastatic disease. Indeed, there were dramatic differences in OS between the two studies: median OS was 60.7 and 11.4 months for non-V600 and V600 mutant mCRC, respectively, in Jones et al., and 8.1 and 4.6 months, respectively, in Shinozaki et al. It is remarkable, however, that in both studies the median OS of non-V600 patients exceeded that of V600 mutant patients. This finding reinforces the observation that non-V600 BRAF mutations have positive prognostic value when compared to V600 BRAF mutations. This may be due in part to the BRAF V600E mutation conferring stronger proliferative potential in a tumour compared to non-V600 mutants.^6^ However, larger retrospective studies will be needed to validate these findings.

The unique finding in the BREAC study was that none of the patients with non-V600 (0/7) or V600 (0/9) BRAF mutant mCRC experienced a partial response to anti-EGFR antibodies. Response rates were similar in RAS mutant patients: 1/40 (2.5%); but were much higher among patients with WT BRAF and RAS: 30/94 (31.4%). Therefore, accounting for the small sample size, the data from BREAC suggests that non-V600 mutations may also function as negative predictive molecular markers for anti-EGFR treatment.

The majority of non-V600 BRAF mutations in CRC are class III mutations.^7,8^ This class of mutations are different from class I (V600) and class II (non-V600 activating mutations) in that they signal as RAS-dependent constitutive dimers, with impaired kinase activity. Given that class III BRAF mutations maintain RAS-dependence, any upstream RAS activating signal (i.e., from an alternate receptor tyrosine kinase) could render a class III mutant tumour intrinsically resistant to single agent EGFR inhibition— this could explain the lack of response observed in the BREAC cohort. In contrast, there have been case reports of patients with class III BRAF mutations (D594G and G466V) experiencing objective responses to EGFR inhibition plus chemotherapy.^2,8^ There is also preclinical evidence of a class III BRAF mutant (G466V) mCRC patient-derived xenograft model undergoing significant tumour regression in response to single agent cetuximab.^8^ As such, there may be some genetic contexts wherein EGFR is the dominant up-stream RAS activator; in these tumours, class III BRAF mutations would maintain sensitivity to EGFR inhibitors. Indeed, by analyzing a subset of 150 mCRC patients from the entire 403 mCRC patient cohort, Shinozaki et al. may have overlooked some mCRC patients with class III BRAF mutations who derived clinical benefit from EGFR inhibitors.

The findings from the BREAC study suggest that non-V600 and V600E BRAF mutant mCRC are similarly unresponsive to EGFR inhibitors. While this is an intriguing hypothesis, with some molecular rationale, it remains to be seen whether all non-V600 BRAF mutations in mCRC tumours are equally predictive of non-response to EGFR inhibitors. Much larger scale retrospective analyses will be required to definitively address this issue. These studies would be warranted to help further refine the subset of mCRC patients who are the most likely to benefit from EGFR-directed therapies.
